# Editorial: Secondary metabolism and fruit quality

**DOI:** 10.3389/fpls.2022.1072193

**Published:** 2022-11-14

**Authors:** María Teresa Sanchez-Ballesta, Itay Maoz, Carlos R. Figueroa

**Affiliations:** ^1^ Department of Characterization, Quality, and Safety, Institute of Food Science, Technology and Nutrition (ICTAN-CSIC), Ciudad Universitaria, Madrid, Spain; ^2^ Department of Postharvest Science, Agricultural Research Organization, Volcani Institute, Rishon LeZion, Israel; ^3^ Laboratory of Plant Molecular Physiology, Institute of Biological Sciences, Campus Talca, Universidad de Talca, Talca, Chile; ^4^ Millennium Nucleus for the Development of Super Adaptable Plants (MN-SAP), Santiago, Chile

**Keywords:** anthocyanin biosynthesis, fruit ripening, fruit volatiles, gene expression, metabolomics, phytohormones, polyphenolic metabolism, transcription factors

Fruits produce a wide array of secondary metabolites, which perform essential physiological and biochemical functions. These metabolites are essential to interacting with the environment during development and postharvest storage, coping with biotic and abiotic stresses. Secondary metabolites are also of utmost importance in fruit quality from the point of view of consumer acceptability, affecting the color/appearance and the flavor, and their implication in fruit nutritional characteristics. The “*SECONDARY METABOLISM AND FRUIT QUALITY*” Research Topic is a compilation of 12 research articles, one review, and one perspective article covering the most recent advances and integrative insights into genes and compounds from the secondary metabolism related to quality traits of non-climacteric and climacteric fruit, such as apricot, grape, cucumber, kumquat, different citrus species, fig, and eggplant.

The importance of secondary metabolism to the environmental adaptation of grapes has been reflected in the four articles studying pre-harvest treatments (Li et el.; Martins et al.) or climate change (Lu et al.; Gashu et al.) that affect berry quality. Abscisic acid (ABA) plays an important role in non-climacteric fruit quality. Taking advantage of the availability of a new table grape cultivar, ‘Ruiduhongyu’, pink in color and muscatel in flavor, on which the effect of ABA had not yet been explored, Li et al., contributes to our current understanding that exogenous ABA improves fruit quality by mediating the endogenous phytohormone levels in grape. Thus, the endogenous biosynthesis of ABA, auxins, and cytokinins regulated by exogenous ABA was correlated with the improvement in appearance parameters, sugars, anthocyanins, and fatty acids. Pre- and postharvest applications of calcium (Ca) have been increasingly used in grapes to maintain fruit quality, improve fruit firmness, and control total decay. Martins et al. combined integrated metabolomics and directed transcriptomic analysis to study the pre-harvest effect of spraying Ca in white wine grapes (cv. Loureiro) over key genes involved in polyphenol biosynthesis and metabolic profile at harvest time. Authors suggested a specific integrated effect of Ca over biochemical and structural properties of white berries down- and up-regulating the expression of *PG1* and *PAL1*, respectively, leading to increased firmness and higher levels of flavonols, at the expense of fruit size and °Brix.

Climate change modifies environmental conditions for fruit production worldwide. Two research articles studied grapes to understand how climatic changes affect fruit quality, either by exploiting the seasonal climatic variations under the double cropping system (Lu et al.) or differences in temperature and radiation in two experimental vineyards (Gashu et al.). Metabolomic and transcriptomic analysis revealed that under the double cropping system, winter wine and table grape berries presented higher concentrations of different volatiles such as terpenes and norisoprenoids correlated with the accumulation of *VviDXS*s, *VviPSY*s, and *VviCCD*s at the transcriptional level (Lu et al.). However, the metabolomic analysis carried out on ten white wine grape cultivars over three years showed that the effect of radiation and temperature on carotenoid and phenylpropanoid contents during ripening was seasonal and varietal-dependent (Gashu et al.), highlighting the potential of crop plasticity to resist elevated temperatures. Savoi et al. presented a perspective article exploring the universe of omics studies conducted on grapes. The authors presented the first integrated grapevine transcriptomics and metabolomics database developed within the *Vitis* Visualization platform to understand berry quality and secondary metabolism.

The spatiotemporal changes of the secondary metabolism in plants are regulated by a complex network of transcription factors, including AP2/ERF, WRKY, bHLH, bZIP, MYB, and NAC. Two research articles lay the foundation for understanding the role of the bHLH transcription factor family in anthocyanin biosynthesis in fruit. Song et al. identified 118 hypothetical bHLH genes in the fig genome; phylogenetic analysis indicated their classification into 25 subfamilies. Transcriptomic data obtained from the re-mined three fig fruit RNA-seq libraries allowed them to screen *FcbHLH42* as a candidate gene related to anthocyanin synthesis. Indeed, further yeast-two hybrid experiments showed the interaction between FcbHLH42 and anthocyanin synthesis-related proteins and its transient expression in tobacco leaves, which led to an apparent anthocyanin accumulation. However, this does not seem to be a shared role of all bHLHs, since *SmbHLH1* from eggplant was identified as a potential repressor of anthocyanin biosynthesis in eggplant fruit peel (Duan et al.). *SmbHLH1* presented a high identity with *SmTT8*, a *SmMYB113*-dependent positive regulator of anthocyanin-biosynthesis in plants, but results indicated different action mechanisms. Thus, *in vitro* and *in vivo* experiments showed that SmbHLH1 could not interact with SmMYB113, whereas SmTT8 could. In addition, SmbHLH1 inhibited anthocyanin biosynthesis, probably by repressing *SmDFR* and *SmANS* expression.

Integrative studies are a valuable tool for discovering new metabolic pathways, genes, and/or traits. In this special issue, a cucumber (Jo et al.) and kumquat (Ma et al.) fruit quality were investigated using metabolomic and transcriptomic approaches. In the first study, Jo et al. compared the peel and flesh of three different cucumber cultivars (Chuichung, White Dadagi, and Mini), including one (Mini) recently developed in Korea. Results indicated differences between tissues and cultivars. Thus, fruit from the cultivar Mini, which is the smallest, presented higher levels of flavonoids and carotenoids in the pulp. However, these were higher in the peel of Chuichung, which is the largest one. Moreover, the antioxidant activity assays, flavonoid-(*CHS* and *4CL*), and carotenoid-related (*PSY* and *ZDS*) genes followed the same tendency in both cultivars.

By taking advantage of the availability of a spontaneous seedling mutant named ‘Huapi’ from ‘Rongan’ kumquat, having desirable traits such as glossy peel, fewer seeds, and less spicy flavor in comparison to the wild type, Ma et al. investigated the mechanisms related to its unique phenotype. The authors indicated that differences could be explained at the transcriptional and biochemical levels and attributed to the high levels of glycosylated flavonoid and low lysophospholipid accumulation in the mutant. Since this cultivar accumulates large amounts of flavones, Tian et al. carried out a molecular characterization of *FcFNSII-2* to lay the foundations for improving its composition in kumquat fruit. *In vivo* and *in vitro* results confirmed that FNSII-2 could be a good candidate for engineering the pathway since it was able to activate the transcription of structural genes of the flavonoid-biosynthesis pathway, interact with *CHS* and *CHI* genes and convert flavanones into corresponding flavones.

Rey et al. comprehensively addressed a comparative metabolomic and transcriptional study of tocopherol accumulation during fruit maturation of four *Citrus* species, namely orange, mandarin, lemon, and grapefruit. Tocopherol contents were higher in the flavedo and increased in this tissue during maturation, paralleled by the induction of genes *TAT1* and *VTE4*, which regulate homogentisate availability and the conversion of γ- to α-tocopherol, respectively. However, the contents decreased or remained constant in the pulp, reflecting changes in the expression of genes *VTE6*, *DXS2*, and *GGDR*, which regulate phytyl pyrophosphate availability.

Apricot fruits’ aromatic profile consists of a large number of volatile compounds. Among the diverse chemical groups of compounds, esters are considered key odorants that influence flavor quality, similar to other fruits, such as apples. Zhou et al. identified *PaAAT1* as a new candidate gene involved in the biosynthesis of volatiles in apricot fruit. Specifically, transient expression of *PaAAT1* in apricot fruit, together with *in vitro* assays using the recombinant protein, suggest its involvement in the biosynthesis of C_6_ esters during fruit ripening.

There is a growing interest in using rapid, accurate, and cost-effective methods in the food industry and research instead of classic methods, which involve a high amount of chemicals, some of which are often hazardous and costly. Hssaini et al. obtained promising results using the Fourier transform infrared with attenuated total reflectance (FTIR-ATR) spectra coupled with partial least square regression (PLSR) model to predict the amounts of phenolic acids and flavonoids concerning their partitioning between the peel and pulp of fresh figs.

Finally, this Research Topic brings a review article about the molecular biology underlying the branched-chain volatiles (BCVs), essential to the characteristic flavor and aroma profiles of many edible fruits, such as bananas and melons. The authors paid attention to the diversity of BCV compounds identified in edible fruits and reviewed the four general hypotheses concerning the mechanisms of BCV biosynthesis. They also explored whether the regulatory mechanisms known to control similar pathways in mammals could offer potential avenues for altering the BCV content of fruit.

Understanding how secondary metabolism affects fruit quality is necessary to develop novel approaches aiming to reduce losses during pre- and postharvest. We hope readers will find this Research Topic a valuable reference to further explore secondary metabolism regulation and its implication in fruit quality.

## Author contributions

MTS-B wrote the first draft of the manuscript. All authors have revised and approved the manuscript for publication.

## Funding

MTS-B’s work was supported by the CICYT project PID2020-113965RB-I00/AEI/10.13039/501100011033. CRF was supported by the National Research and Development Agency (ANID) – Millenium Science Initiative Program – NCN2021_010 and FONDECYT/Regular 1210941.

## Conflict of interest

The authors declare that the research was conducted in the absence of any commercial or financial relationships that could be construed as a potential conflict of interest.

## Publisher’s note

All claims expressed in this article are solely those of the authors and do not necessarily represent those of their affiliated organizations, or those of the publisher, the editors and the reviewers. Any product that may be evaluated in this article, or claim that may be made by its manufacturer, is not guaranteed or endorsed by the publisher.

